# Systematic optimization for production of the anti‐MRSA antibiotics WAP‐8294A in an engineered strain of *Lysobacter enzymogenes*

**DOI:** 10.1111/1751-7915.13484

**Published:** 2019-09-14

**Authors:** Xusheng Chen, Shanren Li, Lingjun Yu, Amanda Miller, Liangcheng Du

**Affiliations:** ^1^ Department of Biotechnology Jiangnan University Wuxi Jiangsu 214122 China; ^2^ Department of Chemistry University of Nebraska‐Lincoln Lincoln NE 68588 USA

## Abstract

WAP‐8294A is a group of cyclic lipodepsipeptides and considered as the first‐in‐class new chemical entity with potent activity against methicillin‐resistant *Staphylococcus aureus*. One of the roadblocks in developing the WAP‐8294A antibiotics is the very low yield in *Lysobacter*. Here, we carried out a systematic investigation of the nutritional and environmental conditions in an engineered *L*. *enzymogenes* strain for the optimal production of WAP‐8294A. We developed an activity‐based simple method for quick screening of various factors, which enabled us to optimize the culture conditions. With the method, we were able to improve the WAP‐8294A yield by 10‐fold in small‐scale cultures and approximately 15‐fold in scale‐up fermentation. Additionally, we found the ratio of WAP‐8294A2 to WAP‐8294A1 in the strains could be manipulated through medium optimization. The development of a practical method for yield improvement in *Lysobacter* will facilitate the ongoing basic research and clinical studies to develop WAP‐8294A into true therapeutics.

## Introduction

The constant emergence of multidrug‐resistant bacterial pathogens is a major threat to human health, and nosocomial infections caused by methicillin‐resistant *Staphylococcus aureus* (MRSA) in hospitals have become an especially serious clinical problem. Vancomycin serves as the cornerstone of the treatment of these drug‐resistant Gram‐positive infections in the past several decades, which is regarded as a last resort in treating MRSA infections. Unfortunately, there are significant concerns owing to decreasing susceptibility to this agent among *S. aureus*, and clinical failures in treatment of bacteremia and endocarditis. In the past decade, several new drugs, such as daptomycin, telavancin and ceftaroline, have been approved for the treatment of infections caused by drug‐resistant Gram‐positive pathogens (Choo and Chambers, 2016). However, the occurrence of drug resistance to these antibiotics is not a question of *if* but *when*, and there have been reports on MRSA resistant to daptomycin and telavancin (Nigo *et al*., 2017; Roch *et al*., 2017). Therefore, the discovery of new antibiotics is an urgent and continual need for human health.

**Figure 1:**
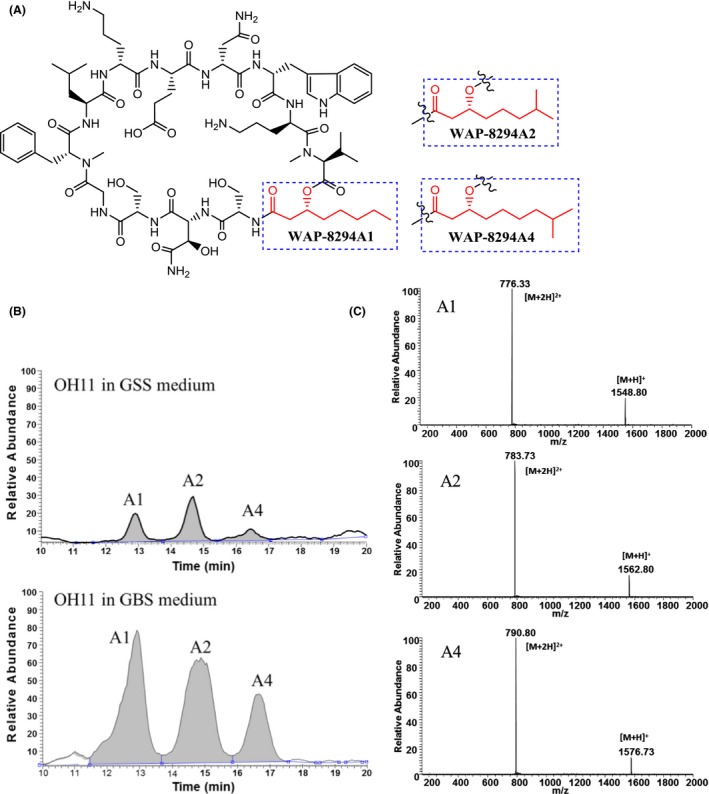
Chemical structure of the main WAP‐8294A compounds isolated from *Lysobacter enzymogenes* OH11 (A), total ion current of extracts from the wild‐type OH11 growing in the starting GSS medium and the optimized GBS medium (B), and mass spectrometry of WAP‐8294A1, A2 and A4 (C).

WAP‐8294A is a family of cyclic lipodepsipeptides containing a 40‐membered macrocycle comprising 12 amino acid residues linked by a small hydroxylated fatty acid. The members of family share the same peptide scaffold and differ by the fatty acid (Fig. 1) (Kato *et al*., 1997, 1998, 2011; Itoh *et al*., 2018). For example, WAP‐8294A2 (Lotilibcin) contains (*R*)‐3‐hydroxy‐7‐methyloctanoic acid and is 14 times more active than vancomycin in anti‐MRSA activity (median effective dose [ED_50_] of 0.38 mg kg^−1^ for WAP‐8294A2 and 5.3 mg kg^−1^ for vancomycin). WAP‐8294A2 is considered the first‐in‐class new chemical entity (depsipeptides) with a high promise for the development of new antibiotics against multidrug‐resistant human pathogenic bacteria. It has been in clinical trials by several biotech companies (Pirri *et al*., 2009). The WAP‐8294A compounds were originally isolated from the environmental Gram‐negative bacterium *Lysobacter staphylocidin*, and WAP‐8294A1, WAP‐8294A2 and WAP‐8294A4 are the main products (Fig. 1) (Kato *et al*., 1997). Later, our group identified the WAP‐8294A biosynthetic gene cluster from *L. enzymogenes* OH11 (Zhang *et al*., 2011). The cluster contains two large nonribosomal peptide synthetase (NRPS) genes that encode a total of 12 modules responsible for the assembly of the 12 amino acids core structure of WAP‐8294A compounds (Zhang *et al*., 2011). In addition, we found several acyl‐CoA ligase genes are involved in the fatty acyl activation and incorporation in WAP‐8294A2 biosynthesis (Chen *et al*., 2015).

While WAP‐8294A2 is a very promising new antibiotic, the drug development has been slow. One of the roadblocks is the very low yield of the WAP‐8294A compounds, which are produced by *Lysobacter* only under certain conditions (Zhang *et al*., 2011). It is vital to develop new methods for yield improvement in *Lysobacter*, so that both the basic research (such as structure−activity relationship and mode of action) and clinical studies can be pushed forward. A sophisticate chemical total synthesis has been reported for WAP‐8294A2, which took more than 30 synthetic steps with a 4% overall yield (Itoh *et al*., 2018). The chemical synthesis of WAP‐8294A2 for the drug development is not feasible at this stage due to the very complex structure. The biological synthesis through fermentation of engineered *Lysobacter* strains under optimized growth conditions remains a feasible approach. Previously, we have tested two biological methods for improving WAP‐8294A production in *L. enzymogenes* OH11. The first was through increasing the expression of a regulator gene in the WAP gene cluster (Wang *et al*., 2013). A TonB‐dependent receptor gene (*orf8*) was put under the control of a constitutive promoter, P_HSAF_, and the resultant *L. enzymogenes* strain gave a twofold higher yield of WAP‐8294A2 than the wild strain. The second strategy was the combined use of CRISPR/dCas9‐mediated gene (*orf1−5*) expression and refactoring of self‐protection gene (*orf6*,* orf 7*,* orf 9* and *orf10*) (Yu *et al*., 2018). This led to an enhanced production of WAP‐8294A by four‐ to ninefold in the engineered strains over the starting strain. Although both strategies increased the WAP‐8294A production in *L*. *enzymogenes* OH11, they require strict culture conditions to maintain the genetic modifications. A more practical method is needed in order to achieve a realistic and satisfactory production of the antibiotics.

The pathway for WAP‐8294A biosynthesis in *L*. *enzymogenes* OH11 is highly complex. The chemical structure of all WAP‐8294A is built from 12 amino acids and a (*R*)‐3‐hydroxy fatty acid (Fig. 1). The WAP biosynthetic gene cluster contains at least 10 genes, and the scaffold of WAP‐8294A is biosynthesized by a complex of 48 domains within the 12 modules of NRPS (Zhang *et al*., 2011). If any of 12 precursor amino acids is short in supply, the final WAP‐8294A production would be affected. Moreover, because the limiting factors in WAP‐8294A biosynthesis are not well understood at present, a global strategy for bioprocess optimization could probably provide a better chance for further improving WAP‐8294A production. In addition, the 12 precursor amino acids of WAP‐8294A are shared by many metabolite pathways in *L*. *enzymogenes*; weakening or deletion of these competition pathways could benefit WAP‐8294A production in theory. In this study, we carried out a systematic investigation of nutritional and environmental conditions in an engineered *L*. *enzymogenes* strain (OH11‐△HSAF, in which the key biosynthetic gene for the main metabolite HSAF in *L*. *enzymogenes* has been deleted) for optimal production of WAP‐8294A. Additionally, we found the ratio of WAP‐8294A2 to WAP‐8294A1 in the strains could be manipulated through medium optimization.

## Results

### WAP‐8294A production in *L enzymogenes*

The WAP‐8294A family consists of more than a dozen of congeners, with WAP‐8294A1, A2 and A4 being the main compounds produced by *Lysobacter* (Fig 1A) (Zhang *et al*., 2011; Yu *et al*., 2018). To perform the yield improvement studies, we need to verify the WAP‐8294A production in the strains. The HPLC analysis of the crude extract from OH11 showed three main peaks, coincident with the standard WAP‐8294A1, A2 and A4 respectively (Fig 1B). The compounds were further confirmed by mass spectrometry, which gave the expected mass for both [M+H]^+^ ion and [M + 2H]^2+^ ion for each of the three main congeners of the WAP family (Fig 1C).

### Semi‐quantitative determination of WAP‐8294A titre using critical dilution assay of inhibition zone

**Figure 2:**
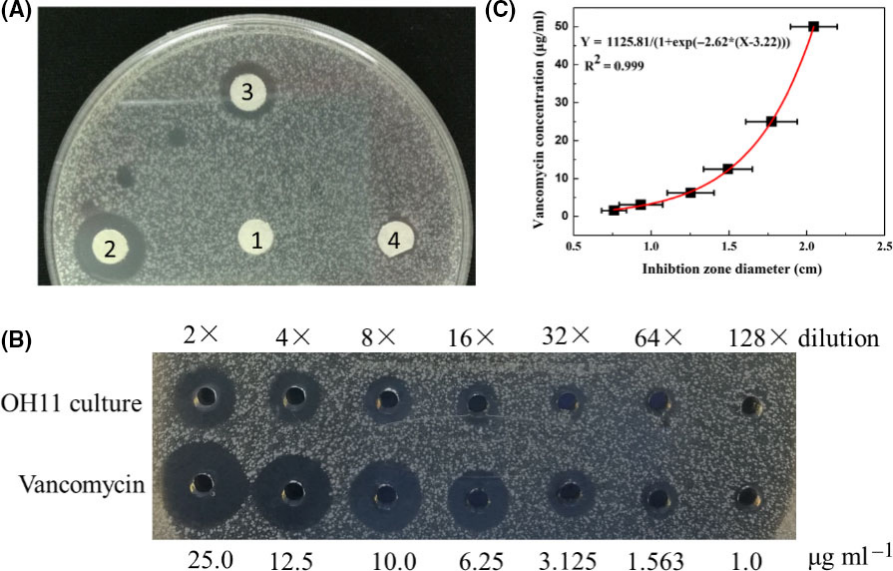
Semi‐quantitative measurement of WAP‐8294A from *Lysobacter enzymogenes* OH11, using the size of inhibition zones of the indicator microorganism *Bacillus subtilis*. A. Inhibitory activity of fermentation broth from (3) the wild‐type strain OH11 (10 μl) and (4) the WAP deletion mutant OH11‐△WAP (10 μl) of *L. enzymogenes* growing in GSS medium, with (1) blank medium (10 μl) and (2) gentamicin (10 μl, 50 μg ml^−1^) as control. B. Inhibition zones of *B. subtilis* on solid LB medium by WAP‐8294A preparations from OH11 (WT) grown in GSS, 10% TSB or R2A media, at 30°C for 72 h, with serial dilutions of vancomycin (starting concentration 50 μg ml^−1^) as control. C. A standard curve of vancomycin concentrations versus inhibition zone diameters on solid LB medium and the derived equation, where X is the inhibition zone diameter, and Y is the concentration of vancomycin. The data were obtained from five repeated experiments.

GSS had been the most commonly used medium for WAP‐8294A production (Kato *et al*., 1997; Zhang *et al*., 2011; Yu *et al*., 2018). However, the yield of WAP‐8294A in the cultures of OH11 strains was very low for a direct quantification by HPLC. It is necessary to develop a relatively fast and reliable method to quantify the amount of WAP‐8294A, before the optimization of the media and growth conditions of the various producing strains. Because WAP‐8294A compounds have a strong antimicrobial activity against *Bacillus subtilis*, we figure a semi‐quantitative method for WAP‐8294A could be developed based on inhibition zone assay for a serial diluted fermentation broth. The fermentation broth of *L. enzymogenes* strain OH11 growing in GSS exhibited a clear inhibition zone, while the mutant with the WAP‐8294A biosynthetic gene deleted, *L. enzymogenes* OH11‐△WAP (Wang *et al*., 2014), did not produce an inhibition zone (Fig 2A). This confirmed that the inhibition zone was formed mainly due to WAP‐8294A production. In addition, the supernatant of strain OH11 produced inhibition zones with different diameters by serial dilution assay (Fig 2B). In parallel, vancomycin also formed inhibition zones with different diameters by serial dilution assay. The relationship between vancomycin concentration and inhibition zone diameter formed an exponential equation with a *R* ^2^ of 0.999, indicating that the equation could explain the relationship with 99.9% reliability (Fig 2C). We consequently used ‘vancomycin equivalent’ to semi‐quantitatively estimate the WAP‐8294A concentration in fermentation broth.

### WAP‐8294A production in strains OH11 and OH11‐△HSAF

**Figure 3:**
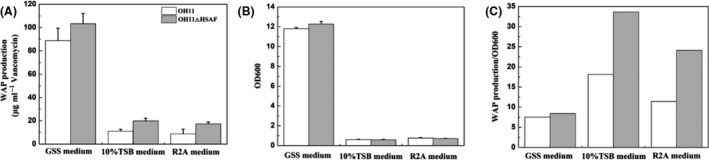
WAP‐8294A yield (A), density of *Lysobacter* cultures grown at 30°C for 72 h (B) and relative yield of WAP‐8294A based on culture density (C). The data were from three replicates.

Using the semi‐quantitative method, we estimated the WAP‐8294A production in the OH11 strains. We used three media, GSS, 10% TSB and R2A, for *Lysobacter* fermentation and compared the total yield of WAP‐8294A, culture density (OD_600 nm_) and the relative yield (WAP/OD_600 nm_) between the strains (Fig 3). The WAP‐8294A yield in GSS medium was 3~6 times higher than that in 10% TSB and R2A media. Strain OH11‐△HSAF reached the highest WAP‐8294A yield of 126.78 μg vancomycin equivalent per ml in GSS medium (Fig 3A). In addition, GSS medium gave the highest cell density (Fig 3B), but resulting in a low relative yield (Fig 3C). The other two media gave a higher relative yield than GSS, but they produced a significantly lower total yield than GSS. In GSS medium, the time‐courses indicated that the strains grew in a similar density in the 72‐h course and strain OH11‐△HSAF gave a higher yield than strain OH11 (Fig. S1). Since the goal of this study was to improve the total yield of WAP‐8294A compounds, we chose OH11‐△HSAF as the starting strain and GSS medium as the starting medium for yield optimization in the subsequent experiments.

### Optimization of media and growth conditions for WAP‐8294A production

**Figure 4:**
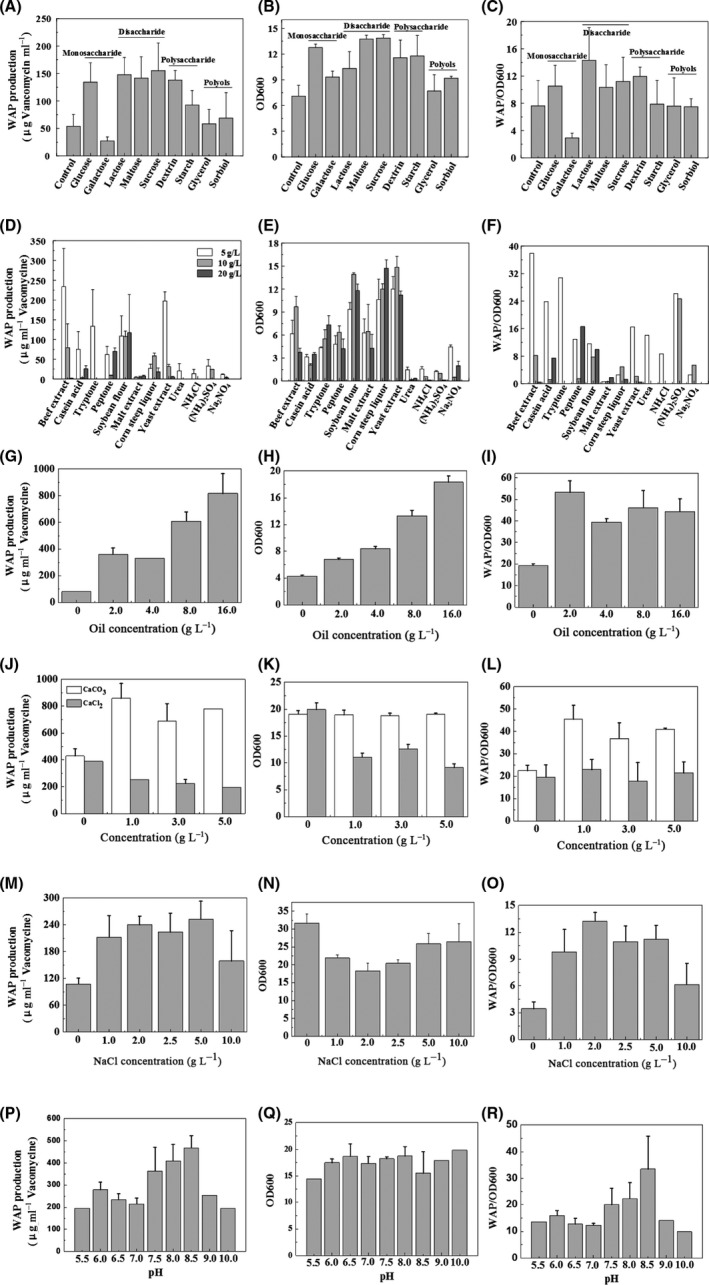
Effects of medium component and pH on WAP‐8294A production, cell density and relative yield of WAP‐8294A of *L. enzymogenes* OH11‐△HSAF. The data were from three replicates.

The effects of monosaccharides (glucose and galactose), disaccharides (lactose, maltose and sucrose), polysaccharides (dextrin and starch) and polyols (glycerol and sorbitol) on WAP‐8294A production and cell growth were evaluated at the concentration of 20 g l^−1^. The results showed that disaccharides were favourite carbon sources for WAP‐8294A production and cell growth by strain OH11‐△HSAF, while polyols were disfavoured by this strain (Fig. 4A–C). Interestingly, this strain could utilize polysaccharides for cell growth and WAP‐8294A production. As shown in Fig. 4A, glucose, lactose, maltose, sucrose and dextrin gave the highest level of WAP‐8294A production, in the range of 130–155 μg vancomycin equivalent per ml. In contrast, galactose gave the lowest WAP‐8294A production, which was even lower than the control (without a carbon source). Moreover, glucose, maltose and sucrose sustained the highest cell growth, OD_600 nm_ ~13 (Fig. 4B). For the relative yield, glucose, lactose, maltose, sucrose and dextrin exhibited a higher WAP/OD_600 nm_ than other carbon sources. Among them, lactose achieved the highest relative yield (Fig. 4C). Glucose also achieved a high WAP‐8294A yield, a high cell growth and a high relative yield among the 9 carbon sources (Fig. 4A–C). Considering glucose is a common carbon source used in fermentation industry, we chose glucose as the optimal carbon source in the modified medium (GBS) for WAP‐8294A production in strain OH11‐△HSAF.

To screen for an optimal nitrogen source for WAP‐8294A production, we tested nine organic nitrogen sources (beef extract, casein acid, tryptone, peptone, soybean flour, malt extract, corn steep liquor, yeast extract and urea) and three inorganic nitrogen sources (NH_4_Cl, (NH_4_)_2_SO_4_ and Na_2_NO_3_) at the concentrations of 5, 10 and 20 g l^−1^ (Fig. 4D–F). Strain OH11‐△HSAF barely grew in GSS medium without a nitrogen source and did not produce any WAP‐8294A (data not shown). All the investigated nitrogen sources, except soybean flour, achieved the highest WAP‐8294A yield when the concentration of the nitrogen source was at 5 g l^−1^; a higher concentration actually gave a lower yield (Fig. 4D). Overall, 5 g l^−1^ beef extract, 5 g l^−1^ yeast extract, 5 g l^−1^ tryptone and 20 g l^−1^ soybean flour gave the highest WAP‐8294A production of 233.8, 197.6, 133.7 and 117.1 μg vancomycin equivalent per ml respectively. Among the inorganic nitrogen sources, (NH_4_)_2_SO_4_ achieved the highest WAP‐8294A yield of 32.5 μg vancomycin equivalent per ml. These results indicated that nitrogen source plays a crucial role in WAP‐8294A production, and the WAP‐8294A yield depends on the nitrogen source's type and concentration. As for cell growth, the organic nitrogen sources (except for urea) were significantly better than the inorganic nitrogen sources (Fig. 4E). OD_600 nm_ of 14.85, 14.71, 13.93 and 9.68 was obtained from the medium containing 10 g l^−1^ yeast extract, 20 g l^−1^ corn steep liquor, 10 g l^−1^ soybean flour and 10 g l^−1^ beef extract respectively. However, the highest WAP/OD_600 nm_ was obtained at 5 g l^−1^ beef extract among the nine organic nitrogen sources, while (NH_4_)_2_SO_4_ gave the highest WAP/OD_600 nm_ at 5 g l^−1^ among the three inorganic nitrogen sources (Fig. 4E). Next, we tested a combined use of beef extract (5 g l^−1^) and (NH_4_)_2_SO_4_ (5 g l^−1^) as the organic and inorganic nitrogen sources, respectively, for WAP‐8294A production in strain OH11‐△HSAF. Unexpectedly, the addition of (NH_4_)_2_SO_4_ into the medium containing beef extract seriously inhibited the cell growth and WAP‐8294A production (Fig. S2). WAP‐8294A was barely detectable when the concentration of (NH_4_)_2_SO_4_ was over 5 g l^−1^.

Previous studies have shown that soybean oil is an important component of media for WAP‐8294A biosynthesis (Kato *et al*., 1997; Zhang *et al*., 2011; Yu *et al*., 2018). To optimize the media, we tested the effect of different concentrations (0–16 g l^−1^) of soybean oil in the media on WAP‐8294A yield and cell growth (Fig. 4G–I). The results showed that soybean oil generally exhibited a positive effect on both WAP‐8294A yield and cell growth. The medium containing 16 g l^−1^ soybean oil produced eight‐ to ninefold more WAP‐8294A than the medium without any soybean oil (Fig. 4G). However, when soybean oil concentration was over 16 g l^−1^, it became difficult to extract and prepare WAP‐8294A from the viscous fermentation broth. Therefore, soybean oil at 16 g l^−1^ was chosen in the modified medium (GBS) for WAP‐8294A production.

The effect of salts on WAP‐8294A production had not been previously investigated. We tested the effect of calcium salts (CaCO_3_ and CaCl_2_) on WAP‐8294A production and cell growth (Fig. 4J–L). The result showed that CaCO_3_ is generally a better calcium source than CaCl_2_ for WAP‐8294A production and cell growth, and the addition of CaCl_2_ into the media actually had a harmful effect. CaCO_3_ at 1.0 g l^−1^ achieved the highest WAP‐8294A production and was used in the modified medium (GBS). Next, we checked the effect of NaCl (Fig. 4M–O). While it had a clearly positive effect on WAP‐8294A production, NaCl exhibited little effect on cell growth in the modified medium. Therefore, 1.0 g l^−1^ NaCl was chosen and used in the modified medium (GBS) for WAP‐8294A production.

Finally, we investigated the effect of initial pH of the media on WAP‐8294A yield and cell growth. While the cell density was not significantly affected by pH in the range of 5.5–10.0, the WAP‐8294A yield was clearly at the highest when pH was in the range of 7.5–8.5 (Fig. 4P–R). WAP‐8294A yield reached 468.5 μg vancomycin equivalent per ml when the GBS medium was at pH 8.5.

### WAP‐8294A production under the optimal conditions

**Figure 5:**
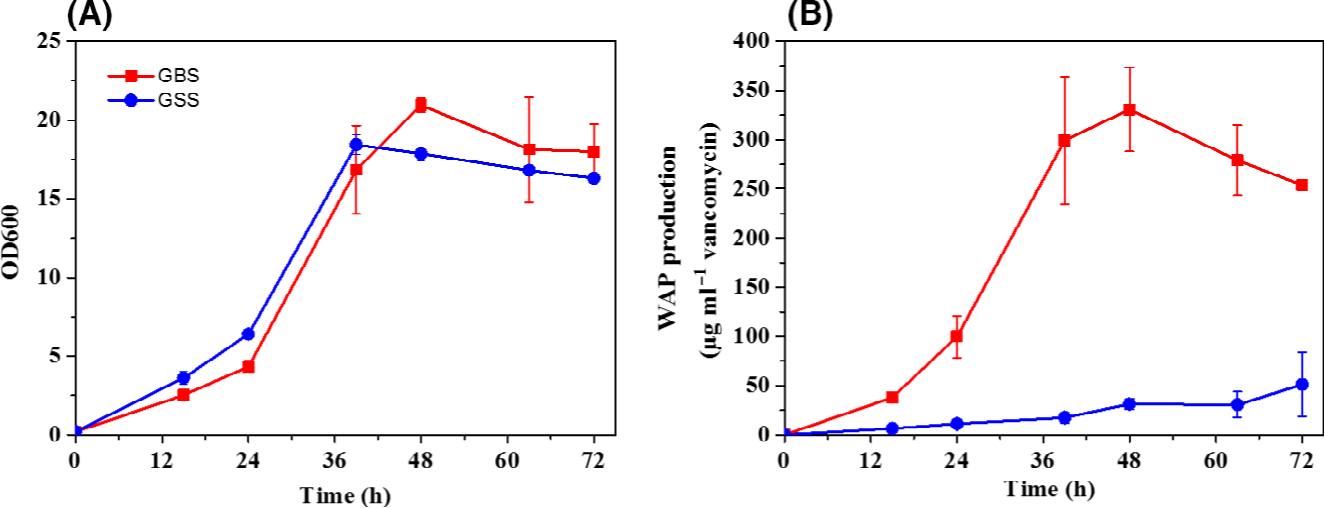
Time‐courses of cell density (A) and WAP‐8294A production (B) by *L. enzymogenes* OH11‐△HSAF in GSS medium (blue curve) versus GBS medium (red curve) in small cultures (50 ml volume in 250‐ml flasks) under the optimized conditions. The data were from three replicates.

**Figure 6:**
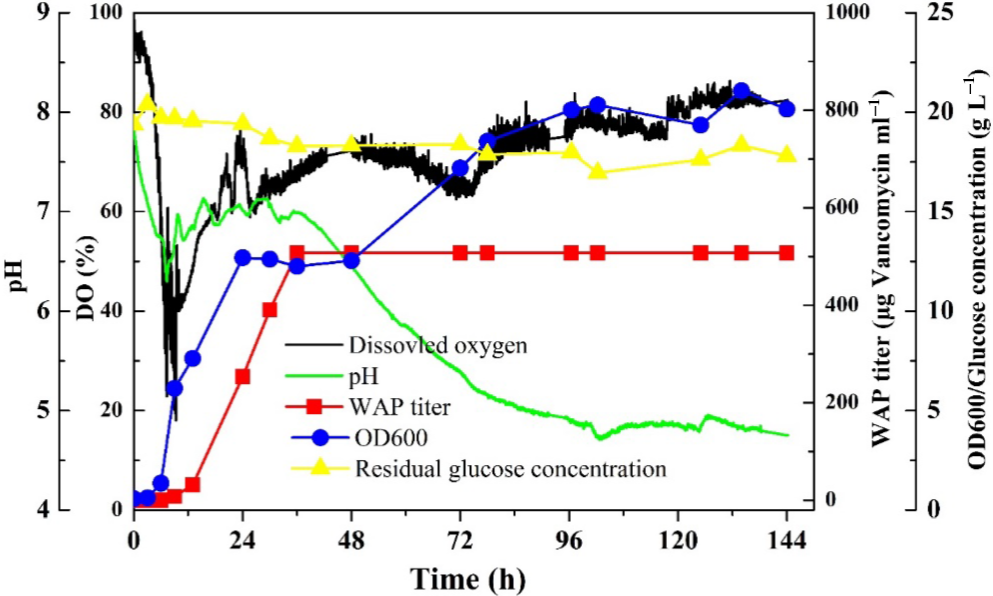
Time‐courses of WAP‐8294A production and culture parameters of *L. enzymogenes* OH11‐△HSAF grown in GBS medium in a large‐scale culture (14 L in a fermenter).

With the development of GBS medium, we scaled up the WAP‐8294A production and cell growth of *L. enzymogenes* OH11‐△HSAF using a fermenter. In standard 250‐ml flasks, the time‐course of WAP‐8294A production was dramatically different in the starting medium (GSS) and the optimized medium (GBS), although the profile of cell density was similar (Fig. 5). WAP‐8294A yield in GBS was significantly increased, reaching 330.6 μg vancomycin equivalent per ml at 48 h, which was over 10‐fold higher than that in GSS medium. In the scale‐up 14‐litre fermenter, the maximum WAP‐8294A yield was achieved by 507.4 μg vancomycin equivalent per ml in GBS at 36 h (Fig. 6). The result indicates that the improvement of WAP‐8294A yield in GBS medium is scalable in strain OH11‐△HSAF. Interestingly, strain OH11‐△HSAF exhibited diauxic growth in GBS medium during the 144‐h fermentation, and the maximum OD_600 nm_ reached 20.1 at 96 h. The dissolved oxygen (DO) of fermentation broth decreased from the initial fermentation until 10th h and then gradually increased and remained around 70% until the end of fermentation. In addition, the pH was kept at around 7.0 before 36 h and afterwards decreased to 4.7 at 96 h and maintained until the end of fermentation.

### Profile change in WAP‐8294A congeners in different conditions

**Figure 7:**
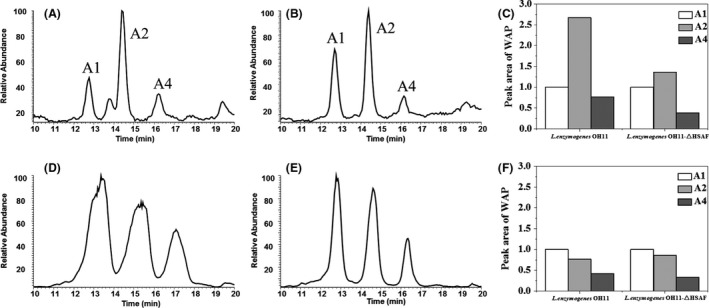
Profiles of the three main congeners of WAP‐8294A from *L. enzymogenes* OH11 and *L. enzymogenes* OH11‐△HSAF grown in GSS medium versus in GBS medium. The compounds were extracted from the cultures using n‐butanol/ethyl acetate (1/1, vol) containing 0.05% TFA. A. Total ion current of the extract from OH11 grown in GSS; (B) total ion current of the extract from OH11‐△HSAF grown in GSS; (C) relative peak area of WAP‐8294A1, A2, A4 in GSS, with WAP‐8294A1 peak area setting to 1; (D) total ion current of the extract from OH11 grown in GBS; (E) total ion current of the extract from OH11‐△HSAF grown in GBS; (F) relative peak area of WAP‐8294A1, A2, A4 in GBS, with WAP‐8294A1 peak area setting to 1.

Among the WAP‐8294A family, WAP‐8294A2 is the predominant member produced by *Lysobacter* strains under standard conditions (Fig. 1) (Kato *et al*., 1997; Zhang *et al*., 2011; Yu *et al*., 2018). To investigate whether the optimized conditions in GBS medium would have any effect on the profile of the WAP‐8294A congeners, we used LC‐MS to examine the production of the three main congeners, WAP‐8294A1, WAP‐8294A2 and WAP‐8294A4 (Fig. 7). LC‐MS data showed that the peak area of WAP‐8294A1, WAP‐8294A2 and WAP‐8294A4 from strain OH11‐△HSAF in the optimized GBS medium increased by 8.2‐, 4.9‐ and 6.7‐fold, respectively, when compared to that in the starting GSS medium. Using standard WAP‐8294A2 as a reference, the yield of WAP‐8294A under the optimized conditions was estimated to be approximately 4 mg l^−1^. In addition, the main WAP‐8294A peak was WAP‐8294A2 when the strains grew in GSS medium, which is consistent with previous reports (Kato *et al*., 1997; Zhang *et al*., 2011; Yu *et al*., 2018). Interestingly, the main peak became WAP‐8294A1 when the strains were growing in GBS medium. The change in the WAP‐8294A profile in GBS medium was unexpected but interesting, because this finding suggests that it may be possible to selectively enhance a particular congener through further optimizing the media.

## Discussion

In this work, we systematically examined the culture conditions optimal for WAP‐8294A production in *L. enzymogenes* OH11. Although the WAP‐8294A family had been isolated more than 20 years ago and one family member (WAP‐8294A2, Lotilibcin) has been in clinical studies for about 10 years, the development of WAP‐8294A into new therapeutics to fight against multidrug‐resistant MRSA and other superbugs still has not been realized. One of the roadblocks is related to the supply of the compounds for both research and development. In this work, we attempted to address this problem by developing culture conditions in which WAP‐8294A yield is significantly improved in *L. enzymogenes* OH11.

The regulatory mechanisms for WAP‐8294A are not well understood at present. There are numerous factors that can contribute to the overall yield of the natural products in *L. enzymogenes* OH11. To optimize the culture conditions quickly and effectively, we needed to develop a simple and easy screening method for antibiotic activity in various culture conditions of *L. enzymogenes* OH11. Using *B. subtilis* as the testing organism, we discovered that a reliable co‐relation between the size of inhibition zones and the vancomycin concentration could be established through serial dilutions of vancomycin concentrations. With this relationship, we were able to use vancomycin equivalent to semi‐quantitatively represent the antibiotic activity of WAP‐8294A in the cultures of *L. enzymogenes* OH11. This simple method enabled us to quickly examine the sources and concentrations of nutrients, including the carbon source, the nitrogen source and other supplements such as soybean oil and salts, as well as other growth conditions. With the optimized culture conditions, we were able to improve the WAP‐8294A yield by 10‐fold, reaching 330.6 μg vancomycin equivalent per ml, in small‐scale cultures, while maintaining a high cell density (Fig. 5). Furthermore, we showed that the optimized culture conditions could be used in scale‐up fermentation, which achieved 507.4 μg vancomycin equivalent per ml in a 14‐litre fermentation (Fig. 6).

Additionally, we found an interesting phenomenon about the profile of the WAP‐8294A compounds in the optimized culture conditions. In all previous studies, WAP‐8294A2 is the predominant congener of the family. However, WAP‐8294A1 becomes the major congener when the strains of *L. enzymogenes* OH11 were growing under the modified conditions. WAP‐8294A1 differs from WAP‐8294A2 by one methyl group in the side‐chain fatty acid of the cyclopeptides (Fig. 1). This suggests that the fatty acid precursor for WAP‐8294A1 might be more abundant in the medium, or more efficiently activated and incorporated under the conditions, or both. Further experiments are needed to confirm this idea. Notably, although WAP‐8294A2 is not the predominant congener, the yield of all three compounds, WAP‐8294A1, WAP‐8294A2 and WAP‐8294A4, increased by 8.2‐, 4.9‐ and 6.7‐fold, respectively, under the conditions. It should also be pointed out that this estimation was based on LC‐MS analysis, and the peak area‐based result is in general agreement with the activity‐based (vancomycin equivalent) results for yield improvement (10‐ to 15‐fold increase of the overall activity).

In summary, we have developed a practical culture method for WAP‐8294A yield improvement in *L. enzymogenes* OH11. The method enables to prepare a relatively large quantity of WAP‐8294A compounds in a relatively short time frame. This will greatly facilitate the ongoing efforts in the basic research and clinical studies to develop the potent antibiotic compounds into true therapeutics for treatment of infections.

## Experimental procedures

### Microorganisms and culture conditions

*Lysobacter enzymogenes* OH11 (CGMCC No. 1978) (Qian *et al*., 2009) is the wild‐type strain, from which we previously isolated the WAP‐8294A compounds (Zhang *et al*., 2011). *L. enzymogenes* OH11‐△HSAF is an engineered strain, in which the key HSAF biosynthetic gene (*hsaf‐pks‐nrps*) has been deleted (Wang *et al*., 2014). This strain provides a relatively ‘clean’ background for metabolite isolation, because HSAF is the major natural product constitutively produced by OH11 (Yu *et al*., 2007). The media used for growth of all strains of *L. enzymogenes* OH11 is listed in Table S1.

### Extraction and analysis of WAP‐8294A compounds from *L. enzymogenes* strains

Stock cultures of *L*. *enzymogenes* strains OH11 and OH11‐△HSAF were inoculated to LB medium containing 50 μg ml^−1^ kanamycin and allowed to grow at 30°C for overnight with 200 rpm shaking. A fraction (1%, 0.5 ml) of the seed culture of each of the strains was inoculated into a 250‐ml flask containing 50 ml of one of the three media, GSS, 1/10 TSB or R2A. The cultures were incubated at 30°C for 72 h with 200 rpm shaking. The procedure for extraction and analysis of WAP‐8294A compounds was essentially identical to that reported previously (Yu *et al*., 2018). Briefly, the culture broth was collected and added with 37% HCl to adjust pH to 2.5. A solvent mixture, n‐butanol/ethyl acetate (1/1, vol) containing 0.05% TFA, was added to the broth to extract the compounds, and the organic phase was collected and dried. Finally, methanol (200 μl containing 0.05% TFA) was used to dissolve the residues that contained WAP‐8294A compounds. For qualitative analysis, a 20 μl aliquot of each extracts was analysed by LC‐MS as described previously (Yu *et al*., 2018). For semi‐quantitative analysis, the inhibition zone method was used (Delgado *et al*., 2005; Falzone *et al*., 2017). *Bacillus subtilis* was used as the indicator microorganism for the antibacterial activity of WAP‐8294A compounds, and the size of inhibition zones on plates was used as a measurement of WAP‐8294A concentration, based on a standard curve generated from a serial dilution of vancomycin as a function of the size of inhibition zones. Specifically, *B. subtilis* was incubated in 3 ml LB medium at 37°C for 6 h with shaking. The culture was diluted using LB medium to obtain a bacterial solution with OD_600 nm_ of 0.3. The indicator microorganism solution was mixed with LB medium (50°C) containing 0.8% agar at the ratio of 1:1000, and the mixture was poured into a plate for solidification. Wells of 4 mm diameter were punched on the plates, and each of the wells was added with an aliquot (20 μl) of various WAP‐8294A preparations that were serial diluted by ddH_2_O (twofold to 128‐fold dilutions). Vancomycin was used as a control in the assays, in which a serial dilution of vancomycin was added to wells on the plates in parallel to the WAP‐8294A preparations. Finally, the plates were incubated at 30°C until clear inhibition zones appeared. The diameter of inhibition zones was measured, and the experiments were repeated five times until a relationship between the zone diameters, and vancomycin concentrations was established, which was used to derive a standard curve for semi‐quantitative estimation of ‘vancomycin equivalent’ activity in the WAP‐8294A preparations.

### Evaluation of the effect of individual nutrients and growth conditions on WAP‐8294A production

Strain *L. enzymogenes* OH11‐△HSAF was selected for the study, because this strain consistently produced a higher yield than other strains in the starting GSS medium (Table S1) (Zhang *et al*., 2011). To evaluate the carbon nutrient's influence on WAP‐8294A production, we tested nine different carbon sources, which were used to replace glucose (20 g l^−1^) in the starting GSS medium. A fraction (1%, 0.5 ml) of overnight seed culture of strain OH11‐△HSAF was inoculated into each of the media (50‐ml in 250‐ml flasks) that contained a different carbon source. The flasks were incubated at 30°C for 72 h with shaking at 200 rpm. The WAP‐8294A compounds were extracted from the cultures and measured using the method described above. Similarly, to evaluate the effect of different nitrogen sources on WAP‐8294A production, soybean flour in the starting GSS medium was replaced with one of 12 nitrogen sources, at the concentration of 0, 5, 10 and 20 g l^−1^. Meanwhile, glucose (20 g l^−1^) was used as the carbon source and other nutrients were not changed.

In addition, the effect of different concentrations of (NH_4_)_2_SO_4_ on WAP‐8294A was assessed. Ammonium sulphate, at the concentration of 0, 1, 3, 5 and 10 g l^−1^, was chosen as the inorganic nitrogen source in the modified GSS medium, in which glucose (20 g l^−1^) was used as carbon source, beef extract (5 g l^−1^) was used as organic nitrogen source, and other nutrients were not changed. Similarly, the effect of different concentrations of soybean oil on WAP‐8294A production was evaluated. Soybean oil at the concentration of 0, 2, 4, 8 and 16 g l^−1^ was tested in the same modified GSS medium.

To test the effect of salts, WAP‐8294A production was compared in a modified GSS medium containing either CaCO_3_ or CaCl_2_, at the concentration of 0, 1, 3, 5 and 10 g l^−1^. This modified GSS medium contained glucose (20 g l^−1^), beef extract (5 g l^−1^), soybean oil (16 g l^−1^) and other nutrients in the original GSS medium. Next, different concentrations of NaCl, 0, 1, 2, 2.5, 5 and 10 g l^−1^, were tested for their effect on WAP‐8294A production in a medium containing glucose (20 g l^−1^), beef extract (5 g l^−1^), soybean oil (16 g l^−1^) and CaCO_3_ (1 g l^−1^), with no change in other nutrients in the original GSS medium. From these tests, a new medium, named GBS (Table S1), was developed for optimal production of WAB‐8294A compounds in strain OH11‐△HSAF.

Finally, the effect of initial pH and initial volume of the culture media was tested. GBS medium with an initial pH in the range of 5.5‐10.0 was prepared, and the WAP‐8294A production was measured in each of the conditions. Based on the outcome of pHs, GBS medium with an initial pH of 8.5 was selected for WAP‐8294A production in an initial medium volume varying from 20 to 100 ml in 250‐ml flasks.

## Conflict of interest

None declared.

## Supporting information


**Fig. S1.** Time courses of WAP‐8294A production (A) and cell density (B) of two strains of *Lysobacter* grown in GSS medium at 30°C, and the anti‐*Bacillus* activity of the strains grown in GSS medium at 30°C for 72 h (C). The data were from three replicates.
**Fig. S2.** Effect of (NH_4_)_2_SO_4_ on the WAP‐8294A production (A), cell density (B), and relative yield of WAP‐8294A (C) of *L. enzymogenes* OH11‐△HSAF, using 5 g l^−1^ beef extract as the organic nitrogen source. The data were from three replicates.
**Table S1.** Composition of culture media using in this study.
