# Supplementary material for: Systematic optimization for production of the anti‐MRSA antibiotics WAP‐8294A in an engineered strain of Lysobacter enzymogenes
Source: Microb Biotechnol. 2019 Sep 14;12(6):1430–40. doi: 10.1111/1751-7915.13484 (PMC6801147; doi:10.1111/1751-7915.13484)
Supplement: Supplementary file 1 — Fig. S1. Time courses of WAP‐8294A production (A) and cell density (B) of two strains of Lysobacter grown in GSS medium at 30°C, and the anti‐Bacillus activity of the strains grown in GSS medium at 30°C for 72 h (C). The data were from three replicates. Fig. S2. Effect of (NH4)2SO4 on the WAP‐8294A production (A), cell density (B), and relative yield of WAP‐8294A (C) of L. enzymogenes OH11‐△HSAF, using 5 g l−1 beef extract as the organic nitrogen source. The data were from three replicates. Table S1. Composition of culture media using in this study. [file MBT2-12-1430-s001.docx]

**Supporting Information**

Additional supporting information may be found online in Supporting Information section at the end of the article.

**Fig. S1.** Time courses of WAP-8294A production (A) and cell density (B) of two strains of *Lysobacter* grown in GSS medium at 30°C, and the anti-*Bacillus* activity of the strains grown in GSS medium at 30°C for 72 h (C). The data were from three replicates.

**Fig. S2.** Effect of (NH_4_)_2_SO_4_ on the WAP-8294A production (A), cell density (B), and relative yield of WAP-8294A (C) of *L. enzymogenes* OH11-△HSAF, using 5 g/L beef extract as the organic nitrogen source. The data were from three replicates.

**Table S1.** Composition of culture media using in this study.
