# Supplementary material for: Systematic optimization for production of the anti‐MRSA antibiotics WAP‐8294A in an engineered strain of Lysobacter enzymogenes
Source: Microb Biotechnol. 2019 Sep 14;12(6):1430–40. doi: 10.1111/1751-7915.13484 (PMC6801147; doi:10.1111/1751-7915.13484)
Supplement: Supplementary file 2 [file MBT2-12-1430-s002.pdf]

Supporting Information

Systematic optimization for production of the anti-MRSA antibiotics WAP-8294A in an engineered strain of *Lysobacter enzymogenes*

Xusheng Chen[1,2] • Shanren Li[2] • Lingjun Yu[2] • Amanda Miller[2] • Liangcheng Du[2]

[1]Department of Biotechnology, Jiangnan University, Wuxi, Jiangsu 214122, China

[2]Department of Chemistry, University of Nebraska-Lincoln, Lincoln, NE 68588, USA

[*]Liangcheng Du (Corresponding author)

Phone: 1-402-472-2998; E-mail: ldu3@unl.edu

Table S1. Composition of culture media using in this study.

Medium	Composition (g/L)
GSS medium	glucose, 25.0; soybean flour, 20.0; soybean oil, 4.0; NaCl, 2.5; $CaCO_3$, 5.0; pH 7.2.
10% TSB medium	casein peptone, 1.7; soya peptone, 0.3; NaCl, 0.5; K_2HPO_4, 0.25; glucose, 0.25; pH7.3
R2A medium	yeast extract, 0.5; proteose peptone, 0.5; casamino acids, 0.5; glucose, 0.5; soluble starch, 0.5; Na-pyruvate, 0.3; K_2HPO_4, 0.3; $MgSO_4 \cdot 7H_2O$, 0.05; pH 7.2.
GBS medium	glucose, 20.0; beef extract, 5.0; soybean oil, 16.0; NaCl, 1.0; $CaCO_3$, 1.0; pH 8.5

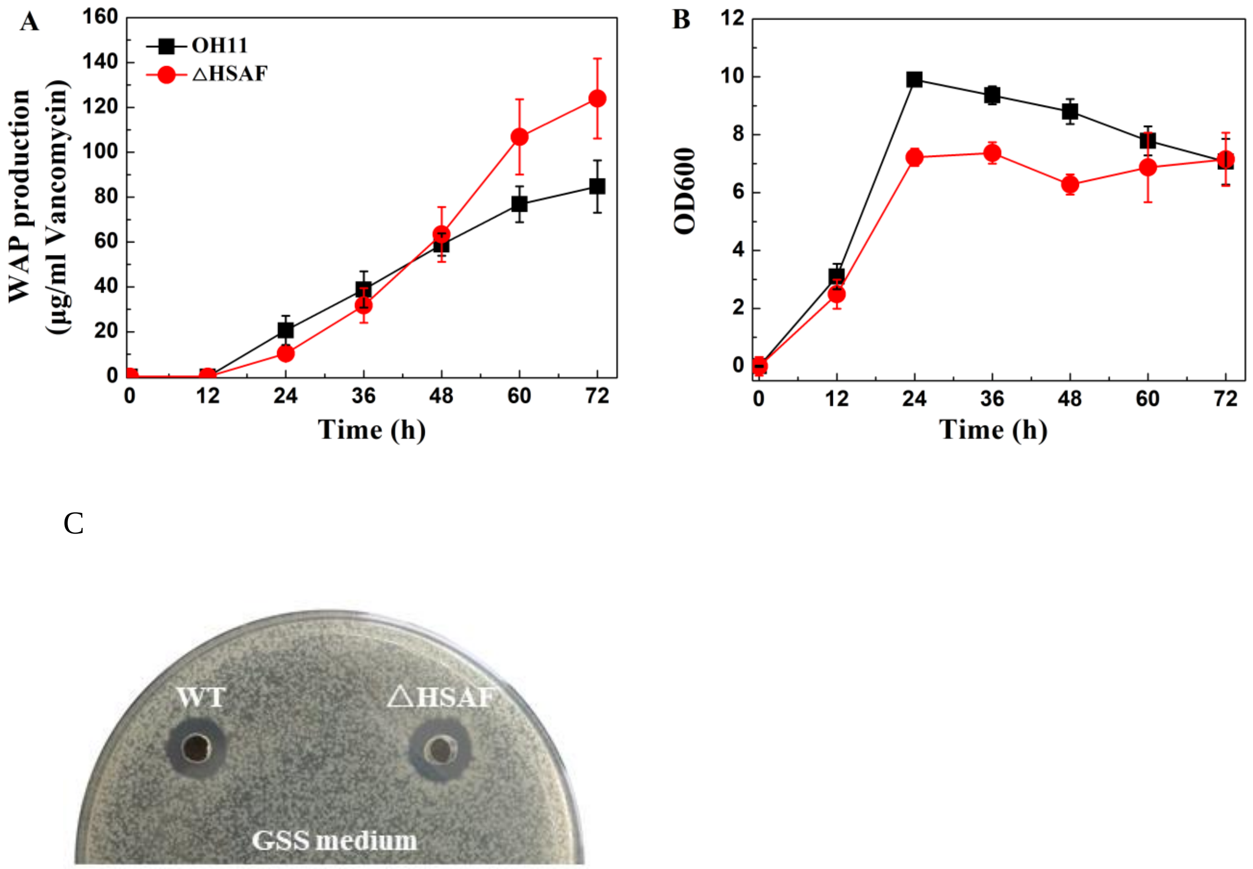

Fig. S1. Time courses of WAP-8294A production (A) and cell density (B) of two strains of *Lysobacter* grown in GSS medium at 30°C, and the anti-*Bacillus* activity of the strains grown in GSS medium at 30°C for 72 h (C). The data were from three replicates.

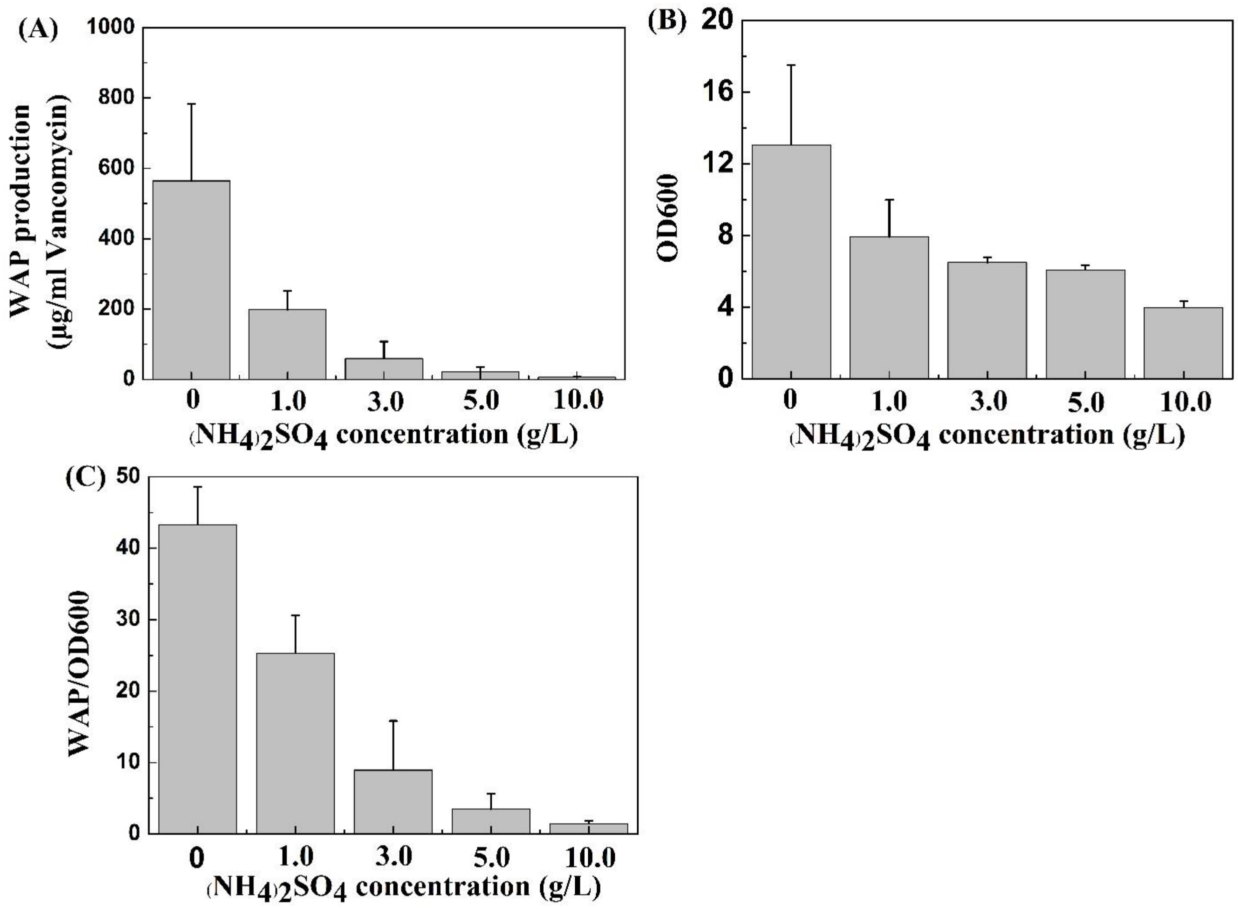

Fig. S2. Effect of $(NH_4)_2SO_4$ on the WAP-8294A production (A), cell density (B), and relative yield of WAP-8294A (C) of *L. enzymogenes* OH11-∆HSAF, using 5 g/L beef extract as the organic nitrogen source. The data were from three replicates.
